# Expression of CC chemokine receptor 7 in tonsillar cancer predicts cervical nodal metastasis, systemic relapse and survival

**DOI:** 10.1038/sj.bjc.6603907

**Published:** 2007-08-07

**Authors:** L Pitkin, S Luangdilok, C Corbishley, P O G Wilson, P Dalton, D Bray, S Mady, P Williamson, T Odutoye, P Rhys Evans, K N Syrigos, C M Nutting, Y Barbachano, S Eccles, K J Harrington

**Affiliations:** 1 Thomas Tatum Head and Neck Unit, St George's Hospital, London, UK; 2 The Institute of Cancer Research, McElwain Laboratories, Sutton, UK; 3 Department of Cellular Pathology, St George's Hospital, London, UK; 4 Head and Neck Unit, Royal Marsden Hospital, London, UK; 5 Statistics Unit, Royal Marsden Hospital, London, UK; 6 The Institute of Cancer Research, Chester Beatty Laboratories, London, UK

**Keywords:** CCR7, chemokine receptor, head and neck cancer, metastasis, survival, tonsil

## Abstract

The aim of this study was to evaluate the expression of CC chemokine receptor 7 (CCR7) in squamous cell cancer of the tonsil with respect to patterns of spread, relapse-free, overall and disease-specific survival. Eighty-four patients with squamous cell cancer of the tonsil were identified. There was a male predominance of 3 : 1 and the median age at diagnosis was 53 (range 35–86) years. The median duration of follow-up was 33 (range 2–124) months. There was a significant association between CCR7 immunopositivity and synchronous cervical nodal metastasis in patients with tonsillar cancer (Spearman's correlation coefficient 0.564; *P*<0.001). Relapse-free (*P*=0.0175), overall (*P*=0.0136) and disease-specific (*P*=0.0062) survival rates were significantly lower in patients whose tumours expressed high levels of CCR7. On multivariate analysis, high-level CCR7 staining predicted relapse-free (hazard ratio 3.0, 95% confidence intervals 1.1–8.0, *P*=0.026) and disease-specific (hazard ratio 10.2, 95% confidence intervals 2.1–48.6, *P*=0.004) survival. Fifteen percent of patients with the highest level of tumour CCR7 immunopositivity relapsed with systemic metastases. These data demonstrated that CCR7 expression was associated with cervical nodal and systemic metastases from tonsillar cancers. High levels of CCR7 expression predicted a poor prognosis.

The development of metastatic disease in loco-regional cervical lymph nodes is a hallmark of squamous cell cancer of the head and neck (SCCHN). Such is the predilection of this disease for lymphatic metastasis that patients may present with pathologically involved cervical nodes at any time during the natural history of the disease (Munro *et al*, 2002). The phenomenon of cervical nodal metastasis from an occult primary mucosal site in the head and neck is well recognised (de Braud and al-Sarraf, 1993). In addition, involved cervical nodes can present synchronously with the primary tumour or metachronously as the first sign of disease relapse.

The presence or absence of lymphatic metastasis is the most important prognostic factor for patients with SCCHN (Layland *et al*, 2005). On the basis of this fact, most patients who are diagnosed as having SCCHN will have radiological investigations such as computed tomography (CT) or magnetic resonance imaging (MRI) in an attempt to identify nodal metastases. Even if these tests suggest that the neck is not involved (clinically node-negative or cN0 disease), patients frequently undergo prophylactic treatment of the neck, either by elective neck dissection or radiotherapy, in an attempt to ablate occult micrometastases. Such additional treatment carries a significant morbidity for the patient. Identification of a panel of biomarkers that would predict the likelihood of nodal metastases would represent a useful tool for patient selection for elective or adjuvant treatment of the neck.

Chemokines are small, secreted proteins with characteristic cysteine motifs in their amino-acid sequences (Rossi and Zlotnik, 2000). Most members of the chemokine superfamily have four cysteines and on this basis they have been classified into four groups (CXC or *α*, CC or *β*, C or *γ* and CXC3 or *δ*) according to the motif displayed by the first two cysteines. Chemokines interact with their cognate receptors which are G-protein-coupled, seven-transmembrane receptors (Gerard and Rollins, 2001). Chemokines were initially shown to be involved in controlling the targeted migration of haematopoietic cells, but more recently they have been implicated in a diverse range of physiological and pathological functions including wound healing, the control of angiogenesis and the development of tumour metastases. Indeed, there has been an evolving interest in the role of chemokines and their receptors in the process of tumour metastasis in recent years. A landmark study clearly demonstrated that breast cancer cells that expressed the chemokine receptors, CXCR4 and CC chemokine receptor 7 (CCR7), were capable of preferentially homing to particular tissues (Muller *et al*, 2001).

CC chemokine receptor 7 is known to be the functional receptor for secondary lymphoid organ chemokine. It acts by influencing the migration of activated dendritic cells to regional lymph nodes. Subsequent studies have demonstrated that CCR7 appears to play an important role in the development of lymph node metastases from oesophageal and gastric cancers (Mashino *et al*, 2002; Ding *et al*, 2003; Yan *et al*, 2004). CC chemokine receptor 7 is also expressed by human adult T-cell leukaemia cells that lodge in lymph nodes (Hasegawa *et al*, 2000). Furthermore, expression of CCR7 by murine melanoma cells has been shown to increase the incidence of lymph node metastasis (Wiley *et al*, 2001).

Therefore, in this study we have evaluated the expression of CCR7 in squamous cell cancer of the tonsil with particular reference to the development of synchronous and/or metachronous cervical nodal metastases and systemic relapse.

## MATERIALS AND METHODS

### Cell lines

Head and neck cancer cell lines were obtained from ATCC (Manassas, VA, USA), from the Institute of Cancer Research/Royal Marsden Hospital, London, UK (O-charoenrat *et al*, 2001) or from the Ludwig Institute, London, UK. All cells were grown in Dulbecco's Modified Eagle's medium (GIBCO-Invitrogen, Carlsbad, CA, USA) supplemented with 10% heated-inactivated fetal calf serum (Harlan Sera-Lab, Loughborough, UK).

### Reverse transcription–PCR

PolyA+ mRNA was isolated using a QuickPrep micro mRNA purification kit (GE Healthcare, Chalfont St Giles, UK) according to the manufacturer's instructions. mRNA was treated with DNase I to avoid DNA contamination, extracted with phenol : chloroform (5 : 1), and precipitated with ice-cold isopropanol and 3 M Na acetate (pH 5.2). The RNA was dissolved in RNase-free water, and the Omniscript reverse transcription kit (Qiagen, Crawley, UK) was used to convert mRNA into cDNA using random hexamer pd(N)6 primers. cDNA was amplified by PCR using RedTaq DNA polymerase (Sigma-Aldrich Co, Poole, UK) and specific CCR7 (Yanagihara *et al*, 1998) or CCL21 primers. Autoclaved double deionised water was run to serve as a negative control. Twenty microlitres of PCR product were electrophoresed in 1.2% agarose gels containing ethidium bromide at 120 V and the PCR products were visualised using a UV transilluminator.

### Western blotting

Cells in monolayer culture were washed twice with ice-cold phosphate-buffered saline (PBS) and lysed on ice with lysis buffer containing 150 mM NaCl, 1 mM EDTA, 50 mM Tris, 1% Triton X-100, 1 mM NaF, 1 mM NaVO_3_, 10 *μ*g ml^−1^ TLCK, 5 *μ*M fenvalerate, 5 *μ*M bpVphen, 1 mM PMSF, protease inhibitor cocktail (1 : 100), phosphatase inhibitor cocktail I (1 : 100) and phosphatase inhibitor cocktail II (1 : 100) (all from Sigma). After incubation on ice for 15 min, lysates were centrifuged at 12 000 r.p.m. for 10 min and the supernatant was harvested. Protein electrophoresis was performed using the NUPAGE Novex Bis-Tris Pre-Cast Gel system according to the manufacturer's instructions (Invitrogen). Electrophoresed proteins were transferred onto PVDF membranes using the NUPAGE transfer system with the XCell II blot module (Invitrogen) according to the manufacturer's instructions. Membranes were blocked with 5% milk in TNT solution (50 mM Tris-HCl pH8.0, 150 mM NaCl, 0.1% Tween 20) overnight at 4°C. After incubation with rabbit anti-CCR7 (Epitomics Inc., Burlingame, CA, USA) and mouse anti-*β*-actin (Abcam Ltd, Cambridge, UK) primary monoclonal antibodies, membranes were washed in TNT and probed with HRP-conjugated secondary antibody at room temperature for 1–2 h. The protein of interest was detected using ECL or ECL plus chemiluminescent Western blotting detection reagent and Hyperfilm ECL (Amersham plc., Little Chalfont, UK).

### Patients

Patients who had been diagnosed with squamous cell cancer of the tonsil during the years 1990–2003 were identified from the head and neck cancer databases of the Royal Marsden and St George's Hospitals. Data on the primary tumour stage and the presence of cervical nodal metastases at the time of diagnosis were obtained from the databases. In addition, data were obtained on subsequent development of cervical nodal or systemic tumour metastases. Archival paraffin-embedded tumour tissue was obtained in each of these cases. Specimens from a total of 84 patients who had been diagnosed with squamous cell cancer of the tonsil in whom adequate follow up data were available were processed and analysed. There was a male predominance of 3 : 1 (63 males and 21 females). The median age at diagnosis was 53 (range 35–86) years. The median duration of follow-up was 33 (range 2–124) months.

### Staging

A summary of tumour and nodal stage at presentation as defined by the International Union Against Cancer TNM classification (1997) is presented in Table 1. As can be seen from these data, the majority of patients presented with node-positive late-stage disease. The distribution of patients according to the American Joint Committee on Cancer staging system was as follows: Stage I (T1N0M0)—3 patients (3.6%); Stage II (T2N0M0)—11 patients (13.1%); Stage III (T3N0M0, T1-3N1M0)—22 patients (26.1%); Stage IVA (T4N0-1M0, T1-4N2M0)—41 patients (48.8%); and Stage IVB (T1-4N3M0)—7 patients (8.3%).

### Treatment

Over the 13-year period spanned by the study, there was some variability in the standard departmental treatment protocols. However, most patients underwent excision of the primary tumour (*n*=63), with or without neck dissection (*n*=39) and post-operative radiotherapy to the tonsil and neck (*n*=59). In two patients, post-operative concomitant chemoradiotherapy (cisplatin 100 mg m^−2^ on days 1 and 29) was administered. Seventeen patients received primary radical radiotherapy and five patients underwent induction chemotherapy (cisplatin 100 mg m^−2^ on day 1, 5-fluorouracil 1000 mg m^−2^ on days 1–4 for two cycles) followed by radical chemoradiotherapy.

### Follow-up

The rates of local control, loco-regional control and survival were calculated using the Kaplan–Meier method. The cause of death was used to estimate disease-specific survival. Overall survival was calculated from the date of diagnosis to the date of last follow-up or death from any cause, and disease-specific survival from the date of diagnosis to the date of last follow-up or death from tonsillar cancer. Relapse-free survival was calculated from the date of treatment to the date of documentation of clinical recurrence or death. The impact of salvage treatment following local, regional or systemic failure is not considered in these results, other than for its contribution to the disease-specific survival. The censor date for analysis was December 2005.

### Immunohistochemistry

Formalin-fixed, paraffin-embedded 3-*μ*m sections were cut and placed on 0.1% poly-L-lysine slides (Sigma), dried for 1 h and stored at room temperature until use. Normal archival human spleen and tonsil tissue specimens were identified as positive and negative controls, respectively. In addition, in many of the tumour specimens, the presence of adjacent normal tonsillar mucosa served as a useful internal negative control. Antigen retrieval was not necessary. Immunohistochemical staining was performed using a standard avidin-biotin peroxidase method. Slides were dewaxed and rehydrated through solutions of xylene, alcohol and water according to standard protocols. Endogenous peroxide was blocked with 0.3% hydrogen peroxide solution for 30 min and the slides were then incubated with anti-CCR7 antibody (Abcam Ltd) for 2 h at room temperature. Anti-CCR7 is a goat polyclonal antibody that was raised against mouse CCR7 by immunising with the synthetic 21-mer DPGKPRKNVLVVALLVIFQVC. It also recognises the human CCR7 protein. Following incubation with anti-CCR7, the slides were rinsed three times in PBS solution and incubated for 2 h at room temperature with biotinylated anti-mouse IgG. Slides were again rinsed three times in PBS solution, and subsequently colorimetric visualisation was achieved by addition of 50 ml 3,3′-diaminobenzidine (BDH Laboratory Supplies, Poole, UK) solution at a concentration of 0.3 mg ml^−1^. Sections were counterstained with Mayer's hematoxylin.

### Scoring of staining

All slides were reviewed by the two pathologists, both with specialist expertise in head and neck cancer, and by Pitkin. The observers were blinded to the identity of the patients and to any details regarding clinical stage, patterns of failure or treatment outcome. The pathologists assessed the adequacy of immunostaining in test slides and in negative and positive control slides, and then selected representative areas of tumour for counting. All three observers counted a minimum of 200 tumour cells and agreed a score for each case (see below). Positive immunostaining for CCR7 was seen predominantly in the cell membrane and also in the cell nucleus; there was some variation in the degree of immunostaining. Tumour cells, where there was only patchy weak staining, were counted as negative; cells with uniformly clear staining, regardless of its intensity, were counted as positive. The following scoring system was devised based on the proportion of tumour cells expressing CCR7. Tumours with <1% of tumour cells expressing CCR7 were scored as ‘−’; tumours with 1–33% of tumour cells expressing CCR7 were scored as ‘+’; tumours with 34–66% of tumour cells expressing CCR7 were scored as ‘++’; and tumours with 67% or more of tumour cells expressing CCR7 were scored as ‘+++’.

### Statistical analysis

The primary aim was to investigate the association between the level of CCR7 staining in the primary tumour and the nodal stage at diagnosis. To assess the strength of this relationship, if any, Spearman's rank correlation coefficient was calculated. Secondary end points included cervical nodal and systemic disease recurrence, relapse-free, disease-specific and overall survival. These were assessed using the Kaplan–Meier method, and differences between the patients with low and high levels of CCR7 staining were compared using the log-rank test. The effect of age, tumour stage and CCR7 staining intensity on the various survival parameters was assessed using Cox regression analysis. Data were updated to December 2005.

## RESULTS

### CCR7 expression is detected by RT–PCR and Western blotting in head and neck cancer cell lines

**Figure 1:**
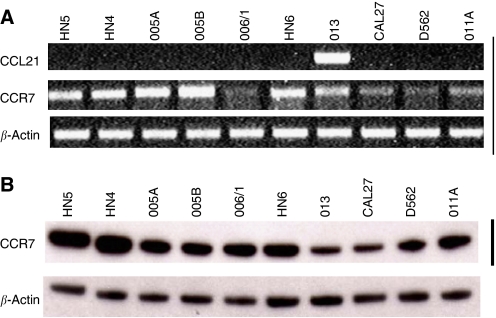
Expression of CCR7 in 10 head and neck cancer cell lines. (**A**). RT–PCR for expression of CCR7 (and one of its ligands CCL21). (**B**) Western blot analysis. In both cases, *β*-actin has been used as a loading control.

CC chemokine receptor 7 was found to be expressed in all cell lines tested with highest levels in 005A, 005B, HN3, HN4 and HN6 (Figure 1). However, the expression of its ligand CCL21 was detectable in only one cell line (013) determined at 40–45 cycles of RT–PCR. The expression of CCL19, another ligand for CCR7, was very low or undetectable in all cell lines tested (data not shown). CC chemokine receptor 7 was also detectable by Western blotting in all cell lines tested. Lower levels were seen in 013 and CAL27 cells and these findings were reasonably consistent with the RNA expression by RT–PCR.

### CCR7 immunostaining correlates with cervical nodal metastasis

**Figure 2:**
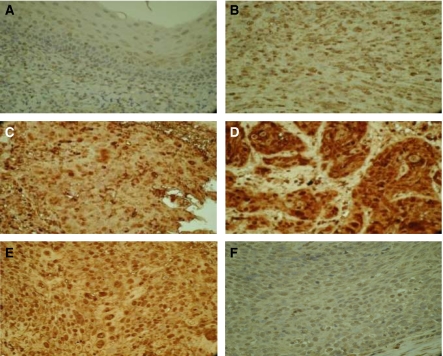
Representative examples of immunostaining for CCR7. (**A**) ‘−’ CCR7 immunostaining in primary SCC tonsil. (**B**) ‘+’ CCR7 immunostaining in primary SCC tonsil. (**C**) ‘++’ CCR7 immunostaining in primary SCC tonsil. (**D**) ‘+++’ CCR7 immunostaining in primary SCC tonsil. (**E**) Positive control CCR7 staining in human spleen. (**F**) Negative control CCR7 staining in normal human tonsil tissue.

**Figure 3:**
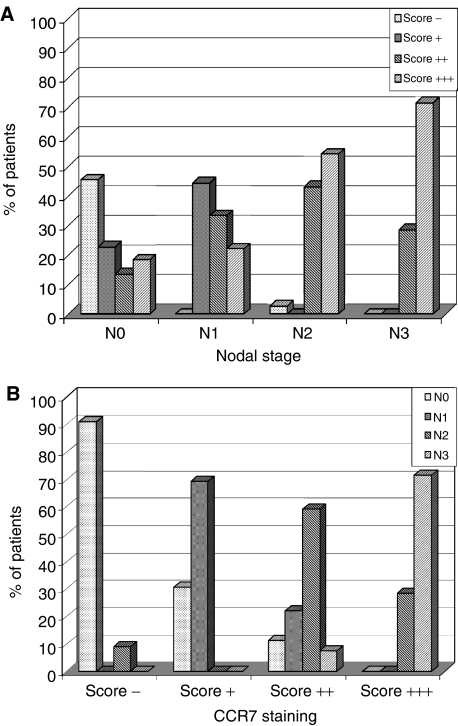
(**A**) CCR7 immunostaining scores correlate with cervical nodal metastasis. (**B**) Rates of cervical nodal metastasis according to CCR7 immunostaining score. Spearman's correlation coefficient is 0.564; *P*<0.001.

Representative examples of tumours in each of these groups are shown in Figure 2A–D. Staining of normal human spleen (positive control) and tonsil (negative control) is shown in Figure 2E and F, respectively. The CCR7 immunopositivity score in the primary tumour is summarised according to the nodal stage at presentation in Table 2. This demonstrates a strong association between CCR7 expression and synchronous nodal metastasis in patients with tonsillar cancer. Therefore, only 1 out of 11 (9.1%) patients with negative CCR7 immunohistochemistry had nodal involvement at presentation, in contrast to 8 out of 13 (61.5%) patients with ‘+’ staining, 24 out of 27 (88.9%) patients with ‘++’ staining and 29 out of 33 (87.8%) patients with ‘+++’ staining (Figure 3A). Similarly, the degree of CCR7 immunopositivity was directly correlated with the extent of nodal metastasis at diagnosis (Figure 3B) (Spearman's correlation coefficient 0.564; *P*<0.001). Therefore, 43 out of 44 (97.7%) patients with N2 or N3 disease had ‘++’ or ‘+++’ immunostaining.

### CCR7 immunostaining correlates with relapse-free survival

**Figure 4:**
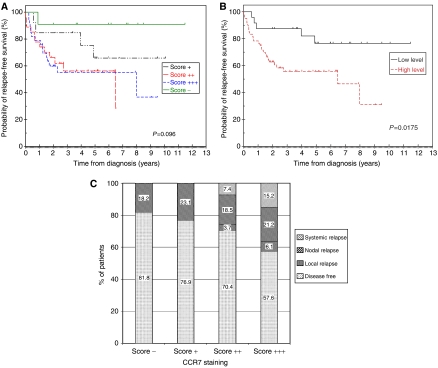
(**A**). Relapse-free survival rates for patients in the four categories (−, +, ++ and +++) of CCR7 immunostaining (*P*=0.096). (**B**) Relapse-free survival rates were significantly different for patients with low (− or +) and high (++ or +++) levels of CCR7 immunostaining (*P*=0.0175). (**C**) Patterns of disease relapse according to CCR7 immunostaining.

Analysis of CCR7 immunostaining in the primary tumour with respect to subsequent disease relapse demonstrated a nonsignificant trend (*P*=0.096; Figure 4A). When the groups were divided into those with low (‘−’ or ‘+’) or high (‘++’ or ‘+++’) levels of CCR7 staining, a statistically significant difference was apparent (*P*=0.0175; Figure 4B). The patterns of relapse are presented in Figure 4C. It is interesting to note that the rates of cervical nodal relapse in the different immunostaining groups were essentially the same at approximately 20%. However, patients with ‘++’ or ‘+++’ staining in the primary tumour were more likely to develop local recurrence or systemic metastases. Indeed, 15% of patients with primary tumours that displayed the highest levels of immunostaining for CCR7 developed systemic metastatic disease. In fact, three patients who presented with cN0 neck disease developed systemic metastases as their first sign of recurrent disease. In two cases, the primary tumour stained ‘+++’ and in the other it stained ‘++’ for CCR7.

### CCR7 immunostaining predicts overall and disease-specific survival

**Figure 5:**
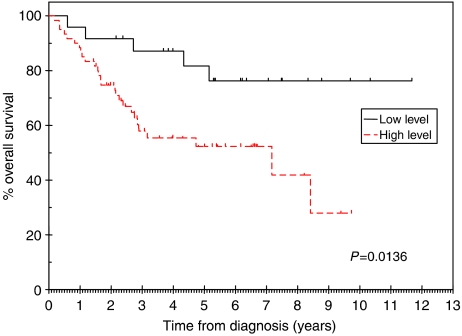
Overall survival rates were significantly different for patients with low (− or +) and high (++ or +++) levels of CCR7 immunostaining (*P*=0.0136).

**Figure 6:**
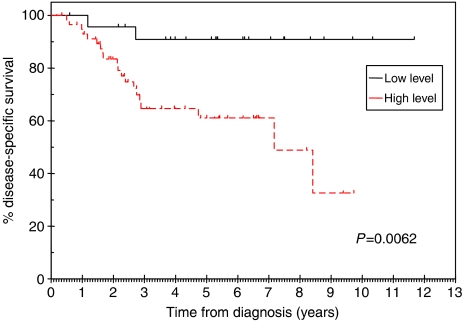
Disease-specific survival rates were significantly different for patients with low (− or +) and high (++ or +++) levels of CCR7 immunostaining (*P*=0.0062).

Patients with primary tumours that displayed high-level (‘++’ or ‘+++’) immunostaining for CCR7 had significantly worse overall and disease-specific survival rates than patients with low level (‘−’ or ‘+’) immunostaining (Figures 5 and 6, respectively). Median overall survival was 7.2 (95% confidence intervals 2.8–11.5) years for the group with high-level immunostaining for CCR7, but was not reached in the group with low-level immunostaining. At 6 years, only 52% of the high-level immunostaining group was alive, compared with 76% of the low-level immunostaining group. If the data were analysed according to disease-specific survival, there was a very significant difference between patients with low- and high-level immunostaining for CCR7 (*P*=0.0062). For patients with high-level immunostaining, the median disease-specific survival was 7.2 (95% confidence intervals 5.8–8.6) years. In contrast, median disease-specific survival was not reached for the patients with low-level immunostaining for CCR7. The 2-year disease-specific survival rates for the patients with low- and high-level immunostaining for CCR7 were 96 and 83%, respectively. The corresponding survival rates at 4 and 6 years were 91 and 65% and 91 and 61%, respectively.

Five patients (6%) died of second primary tumours (four lung, one stomach and one oesophageal cancer), three patients (3.6%) died as a result of post-operative complications and three (3.6%) patients died of intercurrent illnesses.

### Univariate and multivariate analysis confirms CCR7 staining as a significant predictor of relapse-free and disease-specific survival

Cox regression analysis was performed on the data for both relapse-free and disease-specific survival. For relapse-free survival, only T stage (*P*=0.016) and high-level (‘++’ or ‘+++’) CCR7 immunostaining (*P*=0.022) were statistically significant on univariate analysis. Each of these factors remained significant in multivariate analysis. For disease-specific survival, T stage (*P*=0.001), N stage (*P*=0.005) and high-level CCR7 immunostaining (*P*=0.015) were statistically significant on univariate analysis. All of these factors also remained significant in multivariate analysis (Tables 3, 4, 5 and 6).

## DISCUSSION

The development of cervical nodal metastases is a highly significant adverse prognostic feature in patients with SCCHN. However, the molecular basis of tumour cell migration and lymph node metastasis from primary SCCHN is still not fully understood. Recent studies have suggested that normal chemokine signalling pathways that play a central role in lymphoid cell migration and homeostasis can be subverted in SCCHN (and other tumours) to mediate specific patterns of tumour spread. CC chemokine receptor 7 is known to influence the migration of activated dendritic cells to regional lymph nodes and appears to play a similar role in gastric, oesophageal and head and neck cancers (Mashino *et al*, 2002; Ding *et al*, 2003; Wang *et al*, 2004; Yan *et al*, 2004). Mashino *et al* (2002) showed that 66% of gastric carcinoma samples from 64 patients expressed CCR7 and those patients with CCR7-positive tumours had a significantly worse prognosis. Ding *et al* (2003) corroborated these findings with the demonstration that CCR7 mediated lymph node metastasis in oesophageal cancers. Wang *et al* (2004) reported upregulation of CCR7 expression in metastatic tumour lines from 14 patients with SSCHN. Furthermore, Wang *et al* (2005) have shown that CCR7-triggered signalling through the phosphoinositide-3-kinase (PI-3K/protein kinase B (PKB/AKT) signalling cascade is directly involved in chemotaxis and tumour cell invasion of matrigel *in vitro* in two SCCHN cell lines.

In this study, we found a striking correlation between CCR7 immunostaining in primary tonsillar cancers and the presence of cervical nodal metastases at initial diagnosis and subsequent relapse-free, overall and disease-specific survival rates. At presentation, only one patient with a CCR7-negative primary tumour had nodal disease but every patient with ‘+++’ staining had advanced neck disease (N2 or N3). Of course, given the various treatment protocols that were used during the study period and the fact that only 39 of the patients had a pathologically staged neck, there is some uncertainty about the security of the initial diagnosis of cervical nodal disease, although this factor has no effect on the observed relapse-free, overall and disease-specific survival rates. The only patients who underwent neck dissection had clinically or radiologically positive neck disease. The fact that none of the 39 patients who underwent a neck dissection had a pathologically negative neck attests to the accuracy of diagnosis of clinically overt neck disease before surgery. By comparison, no patient with a clinically and radiologically negative neck underwent an elective neck dissection. Therefore, the data on neck stage for these patients were derived exclusively from clinical examination and CT/MRI scans. Therefore, the greatest uncertainty exists for these 22 patients with clinically node-negative necks who did not undergo neck dissection. There is no way of knowing what the pathological neck stage would have been in these patients. However, it is possible to speculate that some of these clinically node-negative patients may have been those who developed nodal or systemic relapse during follow-up. The other 23 out of 45 patients who were staged clinically were recorded as having cervical nodal disease and, given the results of the patients who underwent surgical staging of the neck, it is reasonable to accept that these patients truly had cervical nodal involvement.

The correlation between CCR7 immunostaining in primary tonsillar cancers and the presence of cervical nodal metastases at initial diagnosis may be of interest in selecting patients for elective treatment of the cervical nodes in the setting of a clinically negative neck. Analysis of the data from this study show that by considering ‘−’ staining for CCR7 to mean no nodal metastasis and ‘+’, ‘++’ or ‘+++’ CCR7 staining to mean the presence of nodal metastasis, the test would have a negative predictive value of 90% and a positive predictive value of 80%. Most head and neck oncologists use a threshold risk of cervical nodal metastasis of 20% for recommending elective nodal treatment. Such elective therapy may take the form of surgical dissection or cervical nodal irradiation which, by definition, is unnecessary in most (upto 80%) patients. Both elective neck dissection and elective irradiation expose the patient to potentially serious adverse effects, including accessory or hypoglossal nerve damage, shoulder dysfunction and radiation-induced second malignancy. Therefore, a reliable predictor (or a panel of predictors) of the risk of nodal metastasis would serve as an extremely useful clinical tool for more accurate selection of patients for elective treatment of cervical lymph nodes. CC chemokine receptor 7 immunostaining appears to represent one such potentially useful marker.

CC chemokine receptor 7 immunostaining in the primary tumour appears to predict not only the risk of nodal disease at presentation, but also the risk of subsequent relapse. Twelve patients with high-level CCR7 expression in the primary tumour presented with N0 or N1 disease but went on to develop disease relapse. Analysis of the patterns of recurrence sheds further light on the biological importance of CCR7 expression. Systemic metastatic disease was the first sign of relapse in seven patients (8.3%) and in all cases there was positive immunostaining for CCR7 at presentation. Remarkably, in three of these patients there was no evidence of nodal metastasis either at presentation or in relapse. It can, therefore, be postulated that high-level immunostaining for CCR7 may serve as a predictive marker of systemic metastatic disease. This series of patients received treatment at a time when combined chemoradiotherapy was not widely used in our practice. Therefore, the incidence of systemic metastasis is unlikely to have been influenced by the effect of chemotherapy on subclinical micrometastasis. Despite the fact that adjuvant chemotherapy has not been shown to improve the outcome in head and neck cancer (Pignon *et al*, 2000), the data from this study raise the prospect of selecting high-risk patients for adjuvant trials of new-targeted agents.

CC chemokine receptor 7 immunostaining was also correlated strongly with overall and disease-specific survival. These findings cannot be explained solely in terms of the fact that patients with high-level staining for CCR7 were more likely to have nodal disease at diagnosis (a factor that is known to be strongly predictive of outcome), because CCR7 immunostaining was an independent predictor of disease-specific survival on multivariate analysis. This observation may have been due, at least in part, to the effects of CCR7 on resistance to adjuvant radiation therapy. Since CCR7 signalling in normal immune cells mediates a pro-survival signal through the PKB/AKT cascade (Pilkington *et al*, 2004; Sanchez-Sanchez *et al*, 2004), it is also possible that this pathway may influence outcome in response to treatment in head and neck cancers. Therefore, patients with primary tumours that express high levels of CCR7 may be good candidates for enrolment in studies of new agents that target the CCR7-PI-3K-PKB/AKT axis. It is of note that the improvement in disease-specific survival also translated into a significant increase in overall survival. Therefore, CCR7 immunostaining in the primary tumour may provide an important prognostic marker of overall survival and may be a useful guide to selection of patients for treatment intensification.

In summary, in this study we have shown a strong correlation between CCR7 immunostaining in the primary tumour and the presence of synchronous cervical nodal metastasis. Furthermore, a statistically significant association between CCR7 staining and relapse-free and disease-specific survival was demonstrated. Interestingly, it appears that, in addition to predicting nodal disease, CCR7 may also serve as a predictor of systemic metastasis.

## Figures and Tables

**Table 1:** Summary of TNM stage at presentation in the 84 patients with squamous cell carcinoma of the tonsil

	**N0**	**N1**	**N2a**	**N2b**	**N2c**	**N3**	**Total**
T1	3	7	6	3	0	1	20
T2	11	9	4	10	1	2	37
T3	5	1	3	7	1	2	19
T4	3	1	1	0	1	2	8
Total	22	18	14	20	3	7	84

N0=no nodal metastasis; N1=single ipsilateral cervical nodal metastasis <3 cm in diameter; N2a=single ipsilateral cervical nodal metastasis >3 cm but <6 cm in diameter; N2b=more than one ipsilateral cervical nodal metastases, none >6 cm in diameter; N2c=bilateral or contralateral cervical nodal metastasis, none >6 cm in diameter; N3=cervical nodal metastasis >6 cm in diameter.

**Table 2:** CCR7 immunostaining scores in the primary tumours according to nodal stage at initial disease presentation

	**Score −**	**Score +**	**Score ++**	**Score +++**	**Total**
N0	10 (45.4)	5 (22.7)	3 (13.6)	4 (18.3)	22
N1	0 (0)	8 (44.4)	6 (33.3)	4 (22.2)	18
N2a	0 (0)	0 (0)	7 (50)	7 (50)	14
N2b	1 (5)	0 (0)	8 (40)	11 (55)	20
N2c	0 (0)	0 (0)	1 (33.3)	2 (66.7)	3
N3	0 (0)	0 (0)	2 (28.5)	5 (71.5)	7
Total	11	13	27	33	84

CCR7=CC chemokine receptor 7; N0=no nodal metastasis; N1=single ipsilateral cervical nodal metastasis <3 cm in diameter; N2a=single ipsilateral cervical nodal metastasis >3 cm but <6 cm in diameter; N2b=more than one ipsilateral cervical nodal metastases, none >6 cm in diameter; N2c=bilateral or contralateral cervical nodal metastasis, none >6 cm in diameter; N3=cervical nodal metastasis >6 cm in diameter.

Figures in parentheses represent percentages.

**Table 3:** Univariate analysis of relapse-free survival

**Factor**	***N***	**Hazard ratio (95% CI)**	***P*-value**
*Age*			
Per year	84	1.02 (0.99–1.05)	0.311
			
*Nodes*			
No	24	1	0.546
Yes	60	1.27 (0.58–2.77)	
			
*T stage*			
T1	20	1	
T2	37	1.25 (0.48–3.26)	0.016
T3	19	1.23 (0.40–3.83)	
T4	8	4.72 (1.47–15.09)	
			
*N stage*			
N0	24	1	
N1	16	1.20 (0.42–3.43)	0.248
N2	37	1.08 (0.45–2.57)	
N3	7	2.80 (0.91–8.59)	
			
*CCR7*			
Low (−, +)	24	1	0.022
High (++, +++)	60	3.10 (1.18–8.13)	

**Table 4:** Multivariate analysis of relapse-free survival

**Factor**	***N***	**Hazard ratio (95% CI)**	***P*-value**
*T stage*			
T1	20	1	
T2	37	1.37 (0.52–3.60)	0.521
T3	19	1.13 (0.36–3.51)	0.838
T4	8	4.37 (1.37–13.94)	0.013
			
*CCR7*			
Low (−, +)	24	1	
High (++, +++)	60	3.02 (1.14–8.02)	0.026

**Table 5:** Univariate analysis of disease-specific survival

**Factor**	***N***	**Hazard ratio (95% CI)**	***P*-value**
*Age*			
Per year	84	0.99 (0.95–1.04)	0.751
*Nodes*			
No	24	1	0.762
Yes	60	1.15 (0.46–2.87)	
			
*T stage*			
T1	20	1	
T2	37	1.18 (0.36–3.84)	0.001
T3	19	0.92 (0.21–4.11)	
T4	8	6.14 (1.68–22.39)	
			
*N stage*			
N0	24	1	
N1	16	1.37 (0.42–4.51)	0.005
N2	37	0.64 (0.20–2.04)	
N3	7	4.69 (1.38–15.88)	
			
*CCR7*			
Low (−, +)	24	1	0.015
High (++, +++)	60	6.2 (1.4–26.7)	

**Table 6:** Multivariate analysis of disease-specific survival

**Factor**	***N***	**Hazard ratio (95% CI)**	***P*-value**
*T stage*			
T1	20	1	
T2	37	0.98 (0.27– 3.51)	0.973
T3	19	0.67 (0.15–3.14)	0.615
T4	8	3.77 (0.99–14.28)	0.051
			
*N stage*			
N0	24	1	
N1	16	0.65 (0.17–2.45)	0.521
N2	37	0.22 (0.06–0.80)	0.022
N3	7	1.17 (0.27–5.05)	0.832
			
*CCR7*			
Low (−, +)	24	1	0.004
High (++, +++)	60	10.2 (2.1–48.6)	
